# Functional Outcomes in Orthopaedic Open Wounds Treated With Vacuum-Assisted Closure Therapy: A Prospective Case Series

**DOI:** 10.7759/cureus.54468

**Published:** 2024-02-19

**Authors:** Sunil Gottipati, Bandi Gowtham, Sindhu Chalimeda, Shivani Ravipati, Vishaal P, Kaarvi Khullar

**Affiliations:** 1 Orthopaedics, Great Eastern Medical School and Hospital, Srikakulam, IND; 2 Orthopaedics and Traumatology, Singareni Institute of Medical Sciences, Ramagundam, IND; 3 Medicine, Dr. Pinnamaneni Siddhartha Institute of Medical Sciences and Research Foundation, Vijayawada, IND; 4 Orthopaedics, Stanley Medical College, Chennai, IND; 5 Surgery, Government Medical College, Gondia, IND

**Keywords:** functional outcomes, hospital stay, infection rate, wound healing, vac therapy, orthopaedics, soft tissue injuries, open fractures

## Abstract

Background: Open fractures with extensive soft-tissue damage, significant periosteal stripping, and exposed bone ends pose a significant challenge in orthopaedics. So, we conducted a prospective study that investigated the efficacy of vacuum-assisted closure (VAC) therapy in improving functional outcomes for orthopaedic open wounds.

Methods: A prospective case series was conducted for a period of 18 months at the department of orthopaedics. Seventeen patients with open wounds were included in the study after fulfilling specific criteria. The study involved 17 patients with open wounds, where VAC therapy was applied within 24 hours of admission for 14 patients, and for three patients with superficial infection, thorough wound debridement preceded VAC dressing. Follow-up with patients continued until definitive skin cover procedures were performed.

Results: The majority of cases were attributed to open compound fractures caused by road traffic accidents (82.35%), followed by train traffic accidents (11.76%) and accidental falls (5.88%). The leg (47%) was the most common location of injury, followed by the forearm (23.52%). The average treatment duration was 10.5 days, with an average of 3.3 dressing changes, indicating the efficacy and feasibility of VAC therapy in clinical practice. The average wound size reduction at completion was 15mm, and 15 out of 17 patients achieved successful wound healing. The average time required for forming a uniform granulation bed was 10.5 days, highlighting the efficiency of VAC therapy in promoting tissue regeneration.

Conclusion: Our study findings revealed that there is a significant reduction in the rate of wound infection with the application of VAC therapy, along with a shorter duration for the formation of healthy granulation tissue, rendering the wound suitable for definitive skin cover procedures such as split skin graft and flap cover at an accelerated rate. Additionally, technical challenges associated with applying VAC dressing in the presence of an external fixator were successfully managed through realignment strategies, further underscoring the adaptability and efficacy of VAC therapy in addressing complex wound scenarios.

## Introduction

The management of open wounds in orthopaedics, particularly in cases of fractures with extensive soft tissue damage, periosteal stripping, and exposed fractured ends, has been a significant challenge for both surgeons and patients [1,2]. Traditional methods of wound management, such as daily or alternate-day wound debridement and wet and dry dressing, have been associated with high rates of infection and prolonged healing times [3,4]. However, the emergence of vacuum-assisted closure (VAC) therapy, also known as negative pressure wound therapy, has provided a promising alternative for the treatment of open and complicated wounds in orthopaedics [5-7].

VAC therapy involves the application of negative pressure to the wound through a sealed dressing, which facilitates the removal of exudate, reduces edema, promotes angiogenesis, and leads to the formation of healthy granulation tissue, thereby expediting the wound-healing process [5]. Studies have demonstrated that VAC therapy significantly reduces the incidence of infection, decreases the duration of hospital stay, and minimizes the need for debridement, thereby improving overall patient outcomes [6]. VAC therapy works by applying negative pressure to a wound bed covered by foam. This pressure encourages drainage of exudate and promotes granulation tissue formation, all within a sealed dressing system that collects fluids in a disposable container [7].

Therefore, we aimed to evaluate the functional outcome of open wounds in orthopaedics treated with VAC therapy, with a focus on infection rates, the time required for secondary closure or split skin grafting, and overall wound healing duration.

## Materials and methods

A prospective case series was conducted for a period of 18 months, which included patients admitted to the department of orthopaedics. Seventeen patients with open wounds were included in the study after fulfilling specific criteria. The inclusion criteria are patients with Gustilo-Anderson Type 2, 3a, and 3b compound fractures, acute traumatic soft tissue injuries without bone involvement, and post-operative infected wounds with retained implants. We excluded patients with chronic osteomyelitis, pressure sores, and acute fractures with vascular compromise (Gustilo-Anderson Type 3c). All patients underwent a thorough assessment, including a complete blood count, random blood glucose, renal function tests, CRP, ESR, and X-ray imaging of the affected limb. Additionally, wound cultures were obtained to guide treatment. Prior to the application of VAC therapy, the wounds were assessed for size, area of exposed bone, area of exposed tendons, and presence of any exposed implants. Following thorough debridement and administration of broad-spectrum antibiotics, specific antibiotics were prescribed based on sensitivity profiles. Subsequent dressings involved monitoring of pus culture and sensitivity, wound size, area of bone and tendon exposure, amount of drain collection, duration required for healthy granulation bed formation, and the necessity for skin covering procedures.

**Figure 1:**
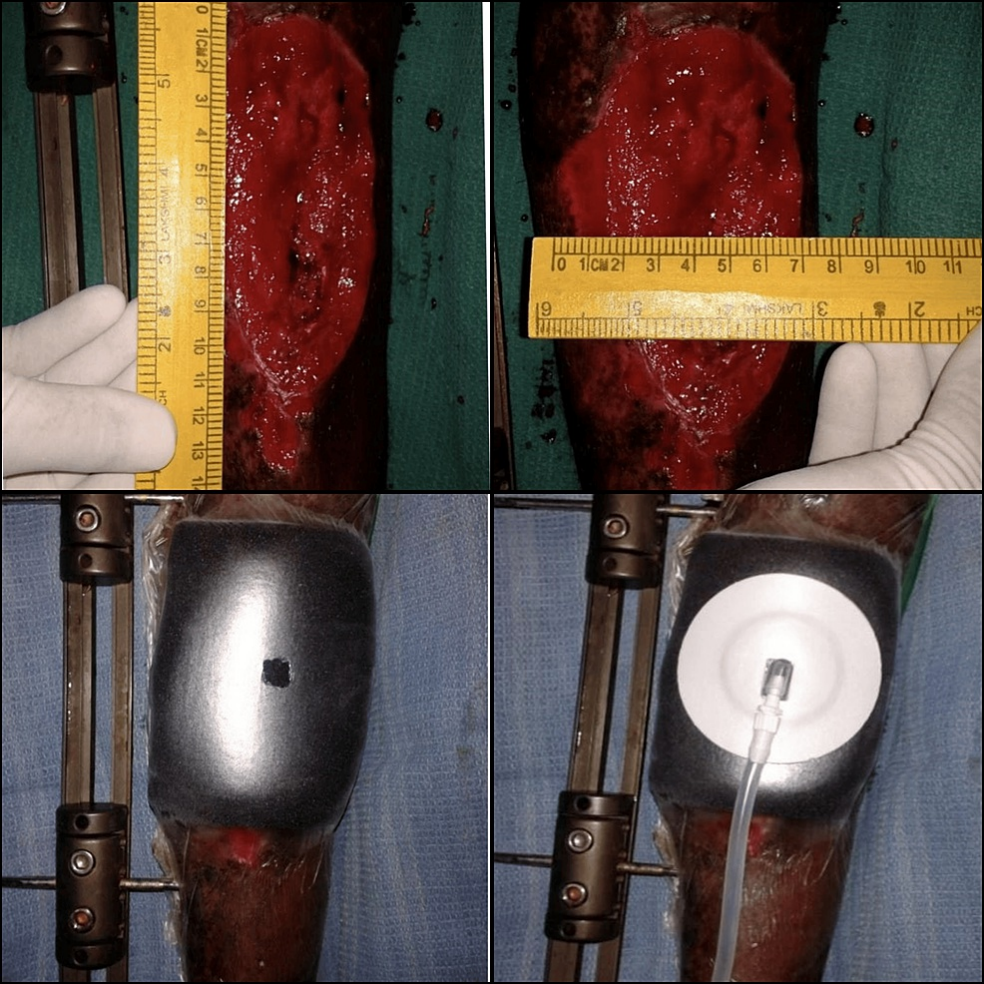
The process and application of VAC dressing for open orthopaedic wounds VAC: vacuum-assisted closure

VAC therapy was applied early, within 24 hours of admission, for 14 patients, while for three patients who developed a superficial infection, thorough wound debridement preceded the application of VAC dressing. Anesthesia techniques varied based on the location of the injury, with spinal anesthesia utilized for lower limb injuries and supraclavicular block for upper limb injuries during debridement, while repeat dressings were performed without anesthesia. The technique for applying VAC dressing involved thorough wound debridement, wound wash, microbiological study, measurement and placement of foam over the wound, covering the wound with a sterile transparent Opsite, insertion of the evacuation tube, connection to a suction machine, and subsequent monitoring. The dressing was removed after three to five days, and skin cover procedures such as split skin grafts and flap covers were considered as needed. Follow-up of patients continued until definitive skin cover procedures were performed (Figure 1).

## Results

Our study involved 17 patients treated with VAC therapy; the mean age of the group was 40.5 years, with the 30-50 age group representing 47% (8) of the cases. Fourteen cases were male, and three were female. The majority of cases were attributed to open compound fractures caused by road traffic accidents (82.35%), followed by train traffic accidents (11.76%) and accidental falls (5.88%). The leg (47%) was the most common location of injury, followed by the forearm (23.52%). The average treatment duration was 10.5 days, with an average of 3.3 dressing changes. We evaluated wound readiness for closure based on the presence of healthy granulation tissue, absence of infection, and adequate skin edge viability. The infection rate was low, with only one case showing infection among the non-infected cases and one case showing infection at the end of treatment among the infected cases, necessitating additional wound debridement and appropriate antibiotics. Demographic details and clinical characteristics of study participants (n=17) are described in Table 1.

**Table 1:** Demographic details and clinical characteristics of the study participants (n=17)

		n	%
Age	21-30	6	35.29
	31-50	8	47.05
	51-60	3	17.64
Gender	Male	14	82.35
	Female	3	17.65
Location of Injury	Forearm	4	23.52
	Thigh	2	11.76
	Leg	8	47.05
	Foot	3	17.64
Size of the wound (cm)	5-10 cm	6	35.30
	10-15 cm	8	47.05
	15-20 cm	3	17.65
Superficial Infection	Non-infected cases	14	82.35
	Infected cases	3	17.65

In our study, open compound fractures of the leg involving Gustilo-Anderson Grades 2 and 3 (47.05%) were the most common presentation. These fractures often expose bone, and in some cases, depending on the location of the injury, tendons can also be affected. Notably, forearm fractures more frequently exhibit tendon exposure. Among the cases with bone exposure, the number of VAC dressing changes ranged from 3 to 4. For tendon exposure, it was primarily three changes, while combined bone and tendon involvement required four changes in most cases. Additionally, two cases with exposed bone and a superficial infection also underwent four dressing changes.

**Figure 2:**
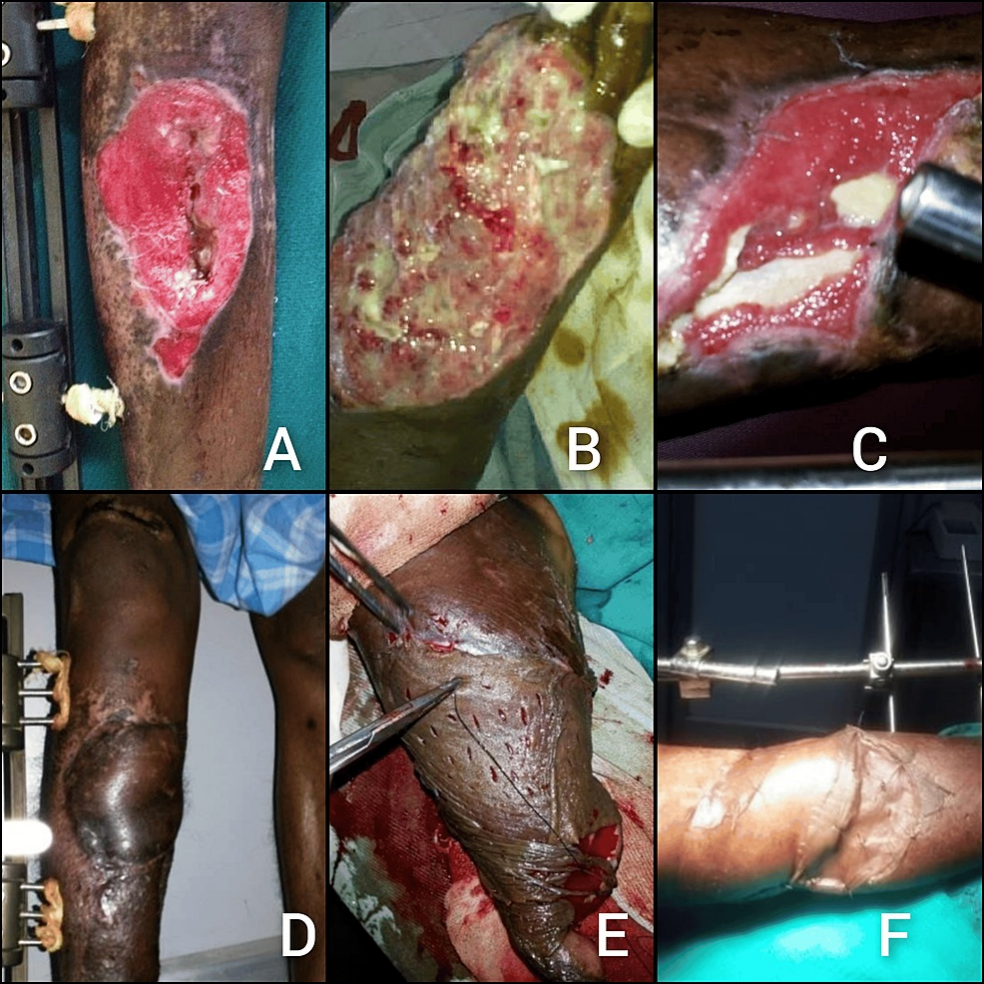
Wounds before and after the application of VAC (A-C) Open orthopaedic wounds before the application of VAC. (D-F) Wounds after the application of VAC VAC: vacuum-assisted closure

The average wound size reduction at completion was 15 mm, and 15 out of 17 patients achieved successful wound healing. Exposed tendons were effectively covered by healthy granulation tissue. Furthermore, the average time required for forming a uniform granulation bed was 10.5 days, highlighting the efficiency of VAC therapy in promoting tissue regeneration. Skin cover procedures, including split skin grafts and flap covers, were performed as needed. We also observed reduced infection and thorough debridement with flap cover in two patients. The average duration of hospital stays was 42 days for the entire study population. There were no complications such as bleeding, overgrowth of granulation tissue over the foam, or deep infections among study participants (Figure 2). The mean follow-up period was 37 days.

## Discussion

Soft tissue management plays a critical role in the management of open compound fractures, with various factors influencing wound healing, including the composition and environment of the wound [1,2]. The primary goals of soft tissue management involve the coverage of soft tissue defects with new granulation tissue and the timely application of a split skin graft or flap cover. Several factors can impact wound healing, such as vascular supply, angiogenesis, matrix protein deposition, local growth factor activity, clearance of necrotic cells, macrophage migration, and enzymatic activity [3,4]. Any deficiency in these factors or their combination can lead to chronic, non-healing wounds. VAC therapy addresses these challenges by promoting increased blood circulation, reducing bacterial burden, and enhancing oxygen supply to the affected soft tissue, thereby facilitating the wound healing process [5-7].

Infection rates

Our study demonstrated a significantly lower infection rate (11.76%) compared to conventional saline dressings in other similar studies, ranging from 27% to 34.7% [8-11]. This suggests that VAC therapy effectively reduces the risk of infection in open fractures.

Granulation tissue formation

VAC therapy facilitated significantly faster formation of a healthy granulation bed (average 10.5 days) compared to saline dressings in other studies. This finding aligns with prior studies in the literature, which established VAC's superiority in accelerating granulation tissue growth [12,13] Microscopic evaluation further revealed increased new blood vessel formation and matrix tissue development under VAC, while saline dressings exhibited inflammatory and fibrous tissues [14]. The uniform negative pressure applied by VAC appears to play a crucial role in promoting healthy granulation tissue formation.

Hospital stay duration

While our study recorded a prolonged hospital stay due to high patient load and limited operating theater availability, it is important to acknowledge that timely definitive management (skin grafting, flap cover, fracture fixation) plays a critical role in minimizing complications and shortening hospitalization. Nevertheless, the literature presents limited data on VAC therapy application in compound fractures. Due to their increased risk of non-union and secondary infection, timely and thorough debridement and skin coverage are paramount for these injuries [15].

Cost-effectiveness

Despite the initial high cost of VAC systems and dressings, compared to saline dressings, VAC therapy may offer long-term cost savings. Saline dressings often require longer wound healing times, multiple debridements, and extended work absences, contributing to higher overall expenses [6,7]. When these factors are considered alongside VAC's reduced morbidity and shorter hospital stays, its cost-effectiveness becomes apparent, potentially freeing up beds for other patients [16,17].

Limitations of the study

The limitations of our study include the non-randomized nature of the research design, which may impact the generalizability of the findings. Additionally, the small sample size and short mean follow-up period could limit the comprehensive assessment of treatment outcomes. Furthermore, the involvement of different surgeons in the definitive management of the wounds, such as split skin grafting and flap cover, introduces potential variability in the treatment approach.

## Conclusions

Our study findings revealed that there is a significant reduction in the rate of wound infection with the application of VAC therapy, along with a shorter duration for the formation of healthy granulation tissue, rendering the wound suitable for definitive skin cover procedures such as split skin graft and flap cover at an accelerated rate. Moreover, we observed a decrease in the number of wound debridements with the use of VAC, with the formed granulation tissue demonstrating a healthy and uniform composition. Importantly, the formation of uniform granulation tissue effectively addressed soft tissue defects, preventing the development of irregular surfaces. Additionally, technical challenges associated with applying VAC dressing in the presence of an external fixator were successfully managed through realignment strategies, further underscoring the adaptability and efficacy of VAC therapy in addressing complex wound scenarios.

## References

[1] Elniel AR, Giannoudis PV (2018). Open fractures of the lower extremity: current management and clinical outcomes. EFORT Open Rev.

[2] Malhotra AK, Goldberg S, Graham J (2014). Open extremity fractures: impact of delay in operative debridement and irrigation. J Trauma Acute Care Surg.

[3] Griffin M, Malahias M, Khan W, Hindocha S (2012). Update on the management of open lower limb fractures. Open Orthop J.

[4] Blease R, Kanlić E (2005). Management of open fractures. Bosn J Basic Med Sci.

[5] Argenta LC, Morykwas MJ (1997). Vacuum-assisted closure: a new method for wound control and treatment: clinical experience. Ann Plast Surg.

[6] Dedmond BT, Kortesis B, Punger K (2007). The use of negative-pressure wound therapy (NPWT) in the temporary treatment of soft-tissue injuries associated with high-energy open tibial shaft fractures. J Orthop Trauma.

[7] Sinha K, Chauhan VD, Maheshwari R, Chauhan N, Rajan M, Agrawal A (2013). Vacuum assisted closure therapy versus standard wound therapy for open musculoskeletal injuries. Adv Orthop.

[8] Gustilo RB, Anderson JT (1976). Prevention of infection in the treatment of one thousand and twenty-five open fractures of long bones: retrospective and prospective analyses. J Bone Joint Surg Am.

[9] Henley MB, Chapman JR, Agel J, Harvey EJ, Whorton AM, Swiontkowski MF (1998). Treatment of type II, IIIA, and IIIB open fractures of the tibial shaft: a prospective comparison of unreamed interlocking intramedullary nails and half-pin external fixators. J Orthop Trauma.

[10] Charalambous CP, Siddique I, Zenios M, Roberts S, Samarji R, Paul A, Hirst P (2005). Early versus delayed surgical treatment of open tibial fractures: effect on the rates of infection and need of secondary surgical procedures to promote bone union. Injury.

[11] Gopal S, Majumder S, Batchelor AG, Knight SL, De Boer P, Smith RM (2000). Fix and flap: the radical orthopaedic and plastic treatment of severe open fractures of the tibia. J Bone Joint Surg Br.

[12] Van Rysselberghe NL, Gonzalez CA, Calderon C, Mansour A, Oquendo YA, Gardner MJ (2022). Negative pressure wound therapy for extremity open wound management: a review of the literature. J Orthop Trauma.

[13] Iheozor-Ejiofor Z, Newton K, Dumville JC, Costa ML, Norman G, Bruce J (2018). Negative pressure wound therapy for open traumatic wounds. Cochrane Database Syst Rev.

[14] Kaushik D, Joshi N, Kumar R, Gaba S, Sapra R, Kumar K (2017). Negative pressure wound therapy versus gauze dressings for the treatment of contaminated traumatic wounds. J Wound Care.

[15] Raj M, Gill SP, Sheopaltan SK (2016). Evaluation of vacuum assisted closure therapy for soft tissue injury in open musculoskeletal trauma. J Clin Diagn Res.

[16] Krtička M, Ira D, Nekuda V, Švancara J, Mašek M (2016). Effect of negative pressure wound therapy on infectious complications in Grade III open fractures (Article in Czech). Acta Chir Orthop Traumatol Cech.

[17] Blum ML, Esser M, Richardson M, Paul E, Rosenfeldt FL (2012). Negative pressure wound therapy reduces deep infection rate in open tibial fractures. J Orthop Trauma.

